# Efficient Internalization of MHC I Requires Lysine-11 and Lysine-63 Mixed Linkage Polyubiquitin Chains

**DOI:** 10.1111/j.1600-0854.2009.01011.x

**Published:** 2009-11-17

**Authors:** Jessica M Boname, Mair Thomas, Helen R Stagg, Ping Xu, Junmin Peng, Paul J Lehner

**Affiliations:** 1 School of Clinical Medicine, Cambridge Institute for Medical Research, University of CambridgeCambridge, CB2 0XY, UK; 2 Department of Human Genetics, Center for Neurodegenerative Diseases, Emory University School of MedicineAtlanta, GA 30322, USA; 3 Current address: Centre for Molecular Microbiology and Infection, Division of Investigative Sciences, Imperial College LondonLondon, SW7 2AZ, UK

**Keywords:** endocytosis, K5, lysine-11, lysine-63, MHC class I, mixed linkage polyubiquitin chains, polyubiquitination, ubiquitin

## Abstract

The downregulation of cell surface receptors by endocytosis is a fundamental requirement for the termination of signalling responses and ubiquitination is a critical regulatory step in receptor regulation. The K5 gene product of Kaposi's sarcoma-associated herpesvirus is an E3 ligase that ubiquitinates and downregulates several cell surface immunoreceptors, including major histocompatibility complex (MHC) class I molecules. Here, we show that K5 targets the membrane proximal lysine of MHC I for conjugation with mixed linkage polyubiquitin chains. Quantitative mass spectrometry revealed an increase in lysine-11, as well as lysine-63, linked polyubiquitin chains on MHC I in K5-expressing cells. Using a combination of mutant ubiquitins and MHC I molecules expressing a single cytosolic lysine residue, we confirm a functional role for lysines-11 and -63 in K5-mediated MHC I endocytosis. We show that lysine-11 linkages are important for receptor endocytosis, and that complex mixed linkage polyubiquitin chains are generated *in vivo*.

In addition to its role in proteolysis, ubiquitin provides a signal for endocytosis and trafficking of internalized receptors (1,2). In the ubiquitin pathway, the highly conserved 76 amino acid ubiquitin molecule is activated by one of two mammalian E1 enzymes, transferred to 1 of around 40 E2 ubiquitin-conjugating enzymes and with the help of one of several hundred E3 ubiquitin ligases, conjugated to the substrate protein (3). The versatility of ubiquitin in post-translational modifications arises from its ability to covalently bind substrate proteins with a variety of forms of ubiquitin. These include one or more single ubiquitins (mono- or multiple monoubiquitination), or polyubiquitin chains linked via one of the seven lysine residues, or the N-terminal methionine, of the acceptor ubiquitin. As alternative ubiquitin chain linkages produce different configurations, they provide a range of signals with potentially different functional outcomes.

Of the many different ubiquitin chain linkages, substrates modified by Lys48-linked polyubiquitin chains are the best characterized, being targeted to proteasomes for degradation. In contrast, Lys63-linked chains provide non-proteolytic signals, as characterized in DNA damage and repair pathways, kinase signalling pathways and endocytosis (4). For other ubiquitin chain linkages, only a limited number of examples of *in vivo* or *in vitro* outcomes are reported. This does not reflect a low frequency of these chain types, as a recent quantitative proteomic analysis in yeast found Lys11-linked chains to be as abundant as Lys48 chains, with all non-Lys48-linked chains being well represented (5). This contrasts with functional data in which only a limited number of examples and outcomes are known for non-Lys48- or Lys63-linked chains.

In higher eukaryotes, receptor internalization usually requires multiple monoubiquitins or a polyubiquitin chain. Where the polyubiquitin chain linkage has been determined, Lys63 conjugates are commonly identified in cell surface internalization, as seen with major histocompatibility complex class I (MHC I) molecules (6), the nerve growth factor receptor (7), aquaporins (8) and the prolactin receptor (9). Furthermore, while it is generally assumed that a polyubiquitin chain contains a single homogeneous chain linkage, this is not necessarily the case, and complexity can be further increased by the potential for assembling ubiquitin chains of mixed linkage. While homotypic chains result from conjugation of ubiquitin through the same lysine linkage (e.g. Lys48 or Lys63), mixed linkage chains result from ubiquitin conjugations that use different lysine linkages for sequential conjugation. This, in turn, can result in a bifurcated (10) or forked chain (11). However, the frequency of mixed linkage chains and their *in vivo* role are unknown.

MHC I molecules sample the protein repertoire of the cell and present peptides to cytotoxic T lymphocytes, alerting the immune system to intracellular pathogens (12). The identification of viral proteins that subvert MHC I antigen presentation highlights the importance of this pathway (13), and provides useful tools to interrogate this process. Three viral immunomodulatory proteins, K3 and K5 of Kaposi's sarcoma-associated herpesvirus and mK3 of the murine gammaherpesvirus-68 were identified by their ability to downregulate MHC I (1,14). Subsequent analysis showed they function as membrane-bound ubiquitin E3 ligases with an N-terminal RING-CH domain (15) required for targeting MHC I for polyubiquitination and degradation (16–18). Both K3 and K5 downregulate MHC I, while K5 is more promiscuous and targets a wide range of immunoreceptors, including CD31, intercellular adhesion molecule type 1 (ICAM-1), B7.2, MHC class I-related chains A and B (MICA, MICB), and activation-induced C-type lectin (AICL) (19–22).

We previously used the K3 viral ligase to study MHC I internalization. K3 causes the rapid Lys63-linked polyubiquitination of MHC I, resulting in the efficient endocytosis and degradation of class I molecules (6). Here, we examine the effect of K5 on MHC I endocytosis and find that internalization of MHC I is less rapid in the presence of K5 than K3, making the K5 system more amenable to study, and allowing a detailed analysis of ubiquitin chain usage. Using a combination of quantitative mass spectrometry (MS), mutant ubiquitins and MHC I with a single cytosolic ubiquitin acceptor, our data show an *in vivo* requirement for complex mixed Lys11- and Lys63-linked polyubiquitin chains in MHC I receptor endocytosis.

## Results

### Increased endocytosis of MHC I following polyubiquitination of the membrane proximal cytosolic tail lysine by K5

**Figure 1:**
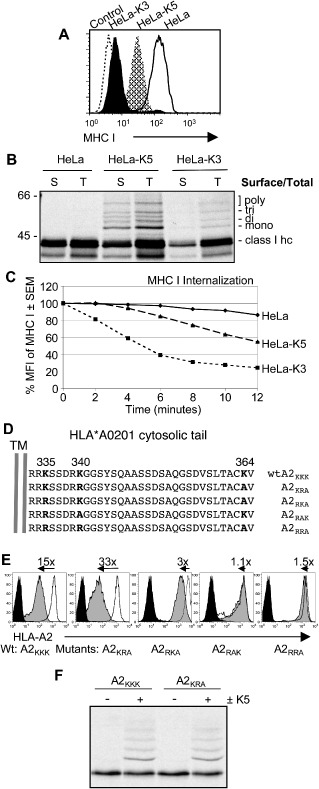
**K5 polyubiquitinates the membrane proximal cytosolic tail lysine residue of MHC I.** A) K3 downregulates MHC I more effectively than K5. Cytofluorometric analysis of MHC I expression in HeLa, HeLa-K3, HeLa-K5 or Cy5 control stained cells. B) K5 causes a predominantly monoubiquitinated MHC I species. Radiolabelled HeLa, HeLa-K3 or HeLa-K5 cells were washed and stained with w6/32 to detect only cell surface MHC I prior to detergent lysis. Postlysis samples were split and processed to detect either surface (S) or total (T) MHC I by w6/32 primary IP and HC10 reIP. C) K3 and K5 increase MHC I internalization in HeLa cells. MHC I internalization was determined in a cytofluorometric-based internalization assay. The change in mean fluorescence intensity (MFI) of the w6/32-reactive MHC I remaining at the cell surface was plotted as a function of time (mean of four replicates ± SEM). D) The cytosolic tail of the wild-type MHC I molecule HLA*A0201 (A2_KKK_) and mutants (lysine residues in bold). E) Cytofluorometric analysis of K5's effects on HLA-A2 and mutants. HeLa cell lines expressing wt and mutant HLA-A2 were transduced with a lentivirus expressing K5 and GFP. Cells were stained with the HLA-A2-specific BB7.2 mAb. Untransduced cells (solid lines), K5 transduced (grey filled) and Cy5 control staining (black filled). The fold downregulation of HLA-A2 by K5 is noted above each histogram. F) HeLa-A2 expressing cell lines ± K5 were radiolabelled and lysed followed by primary IP with BB7.2 and reIP with MR24.

The downregulation of MHC I in HeLa cells is more marked in the presence of K3 (HeLa-K3) than K5 (HeLa-K5) (Figure 1A). Comparison of MHC I immunoprecipitations (IPs) from radiolabelled HeLa, HeLa-K3 and HeLa-K5 cells revealed higher molecular weight bands above the 44 kDa class I heavy chain, which were identified as ubiquitinated MHC I heavy chain species by reprecipitation with both MHC I and ubiquitin-specific antibodies (Figures 1B and S1A). MK3 acts on MHC I in the endoplasmic reticulum (ER), while K3 and K5 affect cell surface MHC I (1). We wanted to ascertain whether any of the ubiquitinated class I species in HeLa-K5 cells were endoglycosidase-H (EndoH) sensitive, suggesting ubiquitination could occur in the ER or in a premedial Golgi compartment. Pulse-chase analysis on radiolabelled HeLa-K5 cells shows that all the ubiquitinated class I is EndoH resistant, suggesting that K5 only affects MHC I in a post-ER compartment (Figure S1B). While the monoubiquitinated MHC I species appears predominant in HeLa-K5 cells, this is not seen with HeLa-K3 cells. This dominance of the monoubiquitinated class I species in HeLa-K5 cells was further exaggerated following IP of cell surface compared with total MHC I (Figure 1B). In HeLa-K3 cells, little ubiquitinated MHC I is detected at the cell surface, reflecting the more rapid endocytosis of MHC I in these cells compared with HeLa-K5 (Figure 1C). Together, these results show that in HeLa-K5 cells, MHC I is readily monoubiquitinated, but this provides a poor internalization signal, suggesting the ubiquitin chain extension in HeLa-K5 cells is relatively inefficient, leaving many monoubiquitinated MHC I molecules stuck at the cell surface.

MHC I heavy chains contain two conserved cytosolic tail lysine residues. We made mutations in these residues in the HLA-A*0201 (HLA-A2) MHC I molecule, which has the two conserved lysines at positions 335 and 340 and an additional lysine at position 364. The conserved (K_340_) lysine residue in the cytoplasmic tail of HLA-A2 is the preferred acceptor site for K3-mediated polyubiquitination (6,18,23). In HeLa-K5 cells, downregulation of the HLA-A2 (A2_KRA_) mutant, expressing only the single membrane proximal lysine (K_335_), was as efficient as downregulation of wild-type HLA-A2 (A2_KKK_) (Figure 1D,E). In contrast, the lysine at position 364 (A2_RAK_) afforded no MHC I downregulation, whereas the conserved lysine at position 340 (A2_RKA_) afforded minimal MHC I downregulation –only twofold higher than a mutant HLA-A2 lacking all cytosolic lysine residues (A2_RRA_) (Figure 1E). The membrane proximal lysine (K_335_) is therefore the dominant ubiquitin acceptor and this was confirmed biochemically, as the single lysine HLA-A2_KRA_ showed an identical polyubiquitin pattern compared with that seen with the wild-type HLA-A2 (Figure 1F). As removal of all cytosolic lysine residues prevents the K5-mediated downregulation of MHC I (Figure 1E), we can exclude contributions from thio- or oxyester ubiquitin chain conjugations via cysteine, serine or threonine residues (24). Thus, K5 differs from K3 in targeting the conserved membrane proximal lysine residue (K_335_) in the cytosolic tail of MHC I for polyubiquitination.

### K5-mediated polyubiquitination of MHC I requires ubcH5 and ubc13

**Figure 2:**
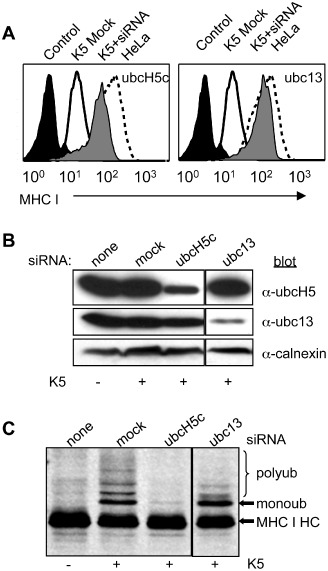
**Polyubiquitination of MHC I by K5 requires ubcH5 and ubc13.** MHC I expression in HeLa-K5 cells is rescued following cellular depletion of ubcH5c and ubc13. A) Cytofluorometric analysis of class I expression 60 h postdepletion of ubcH5c or ubc13 by siRNA. HeLa-K5 + mock siRNA (black line), HeLa (dotted line) HeLa-K5 + siRNA for either ubcH5c or ubc13 (grey filled) and Cy5 control (black filled). B) Immunoblot confirms the siRNA depletion of ubcH5c and ubc13. C) HeLa-K5 cells from (A), depleted of ubcH5c or ubc13, were radiolabelled, lysed and subjected to w6/32 primary IP and HC10 reIP.

K3-induced polyubiquitination of MHC I requires ubcH5 and ubc13 (6). We therefore tested whether K5 also uses these E2 enzymes. MHC I was rescued back to the cell surface in HeLa-K5 cells depleted of ubcH5c and ubc13 by short interfering RNA (siRNA) (Figure 2A). Western blot analysis confirmed effective depletions (Figure 2B). The different effects of the E2 enzymes' depletion were readily distinguishable on biochemical analysis of ubiquitinated MHC I. UbcH5c depletion abrogated K5-mediated MHC I ubiquitination (Figure 2C). In contrast, depletion of ubc13 showed a collapse of the MHC I polyubiquitin ladder to the predominantly monoubiquitinated form.

### Quantitative mass spectrometry identifies Lys11 and Lys63 ubiquitin chain linkages on MHC I from HeLa-K5 cells

**Figure 3:**
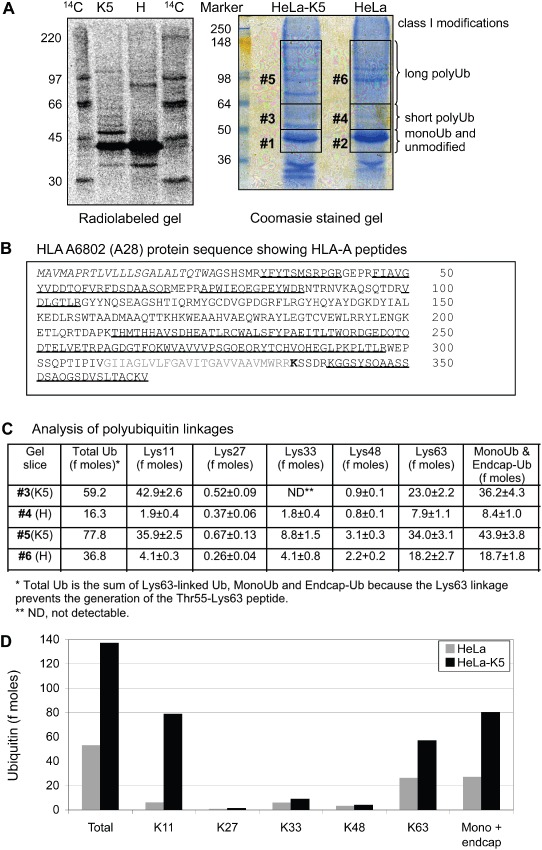
**Quantitative MS identifies Lys11 and Lys63 ubiquitin chain linkages on MHC I from HeLa-K5 cells.** A) Isolated membrane fractions from HeLa and HeLa-K5 cells were solubilized and denatured class I heavy chains were immunoprecipitated with HC10. Following SDS–PAGE and Coomasie staining, the left half of the gel (containing radiolabelled samples) was dried and analysed by phosphorimager. Gel slices (as labelled #1–6), from the right half, were separated, digested and analysed by MS. B) Amino acid sequence of the HLA-A28 class I allele showing peptides retrieved from MS analysis following MHC IIP. Also indicated are the *signal sequence*, transmembrane domain and lysine 335 (K), the dominant ubiquitin acceptor of K5-mediated polyubiquitination. C) Quantification of ubiquitin chain linkages attached to MHC I by the MS-based AQUA method. Peptides were eluted from gel slices #3 and #5 from HeLa-K5 and #2 and #4 from HeLa and quantitated with the addition of eight stable isotope-labelled peptides as internal standards, including all seven –GG peptides and one ubiquitin peptide (Thr55-Lys63). D) Graphical representation of the data in part (C).

To examine the ubiquitin chain linkage required for K5-mediated downregulation of MHC I, we used the ubiquitin-AQUA (absolute quantification of ubiquitin) quantitative MS-based approach (25). The use of eight isotope-labelled internal standards to quantify tryptic peptides, derived from the digestion of monoubiquitin and polyubiquitin chains bound to MHC I, allows a direct determination of the levels of ubiquitinated species. Denatured MHC I was immunoprecipitated from membrane fractions of HeLa and HeLa-K5 cells, with the class I heavy chain-specific (HC10) monoclonal antibody (mAb). Radiolabelled samples were run on the same gel to directly compare Coomasie stains with radioimmune precipitations (Figure 3A), and allow alignment of MHC I heavy chain and the ubiquitinated MHC I species.

Following trypsinization and reverse-phase liquid chromatography (LC) of peptides extracted from the indicated gel slices (Figure 3A), the resulting peptides were analysed by MS/MS. Good coverage of MHC I-derived peptides was achieved. The sequence of HLA-A28, the HLA-A allele in HeLa cells is shown (Figure 3B) and gave 49% coverage (44 peptides) of the HLA-A allele and 46% coverage (24 peptides) of the HLA-B allele. From HeLa-K5 cells, a 2.6-fold increase in total ubiquitin was detected in the region above monoubiquitinated class I, as compared with the corresponding region of HeLa cells (Figure 3A, gel slices #3–6, Figure 3C,D). Polyubiquitin chain linkage analysis (Figure 3C,D) identified an enrichment of Lys11 as well as Lys63 linkages in samples prepared from HeLa-K5 cells with a 13-fold increase in Lys11-linked polyubiquitin chains and a 2.2-fold increase in Lys63-linked polyubiquitin chains compared with HeLa cells. Other ubiquitin chain linkages were either detected at very low levels or not at all (Figure 3C,D).

### Lys63 and Lys11 of ubiquitin are necessary for K5-mediated MHC I endocytosis

As the LC-MS/MS identified an enrichment of Lys11- as well as Lys63-linked ubiquitin chains in the presence of K5, we wanted to identify whether these polyubiquitin chains also played a functional role in K5-mediated MHC I downregulation. To facilitate this, epitope-tagged Lys-to-Arg ubiquitin-green fluorescent protein (GFP) mutants were overexpressed. These mutants are particularly informative as cotranslational cleavage of the C-terminal GFP frees the terminal glycine of ubiquitin for subsequent conjugation (26). The cleaved GFP provides both a quantitative surrogate marker of mutant ubiquitin introduced into the cells, and also allows gating of the ‘GFP high’ population, so that only those cells in which mutant ubiquitin outcompetes endogenous ubiquitin are analysed.

**Figure 4:**
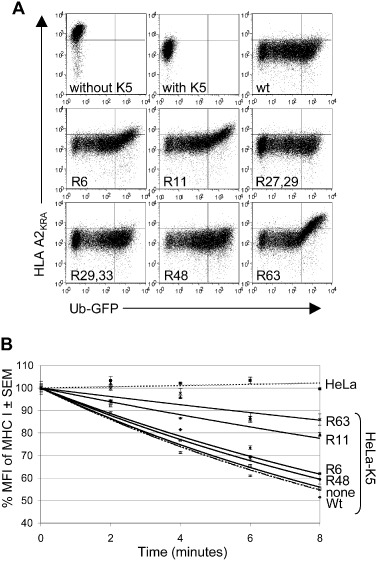
**Lys11 and Lys63 of ubiquitin are necessary for K5-mediated MHC I endocytosis.** A) K5 downregulation of HLA-A2_KRA_ (containing the single cytosolic lysine K_335_) is rescued by overexpression of the ubiquitin mutants R6, R11 or R63. Cytofluorometric analysis of HLA-A2_KRA_ expression in K 5+ cells 3 days post-transduction with ubiquitin mutants as noted. Cells were stained with BB7.2 + Cy5. B) Overexpression of R11 and R63, but not R6 or R48 ubiquitin mutants, interferes with K5-mediated MHC I endocytosis. Internalization of MHC I in HeLa-K5 cells transduced with wt or mutant Ub-GFP 3 days post-transduction as noted. Cells were assayed as in Figure 1C and cells transduced with lentivirus were gated for GFP^high^ expression prior to analysis. Mean of three replicates ± SEM.

When a ubiquitin lysine residue critical for K5 function is mutated, increasing levels of GFP expression on the *x*-axis of the dot plots result in increasing MHC I expression on the *y*-axis, i.e. rescue of MHC I to the cell surface. The effect of ubiquitin mutants was determined on the MHC I molecule HLA-A2_KRA_ (A2_KRA_), which only contains the single K_335_ cytosolic tail lysine residue. We can therefore exclude a role for different ubiquitin chains on different ubiquitin acceptor lysine residues. Overexpression of the ubiquitin mutant R63 Ub-GFP (Lys63 mutated to Arg63) rescues A2_KRA_ surface expression, confirming the requirement for Lys63-linked ubiquitin chains on the single membrane proximal lysine during MHC I downregulation (Figure 4A). Lys63-linked polyubiquitin chains are also required for efficient endocytosis as MHC I internalization was slower in HeLa-K5 cells expressing high levels of R63 Ub-GFP (Figure 4B). Overexpression of the R6 and the R11 Ub-GFP mutants also rescued A2_KRA_ surface expression (Figure 4A). An identical rescue was also seen with R6, R11 and R63 Ub-GFP mutants on total MHC I (Figure S2). Like the R63 mutant, R11 interfered with MHC I endocytosis (Figure 4B), confirming the requirement for Lys11 in K5-mediated MHC I endocytosis, while MHC I internalization was not affected by the R6 mutant.

### Lys6, Lys11 and Lys63 of ubiquitin are necessary and sufficient for K5-mediated MHC I endocytosis

**Figure 5:**
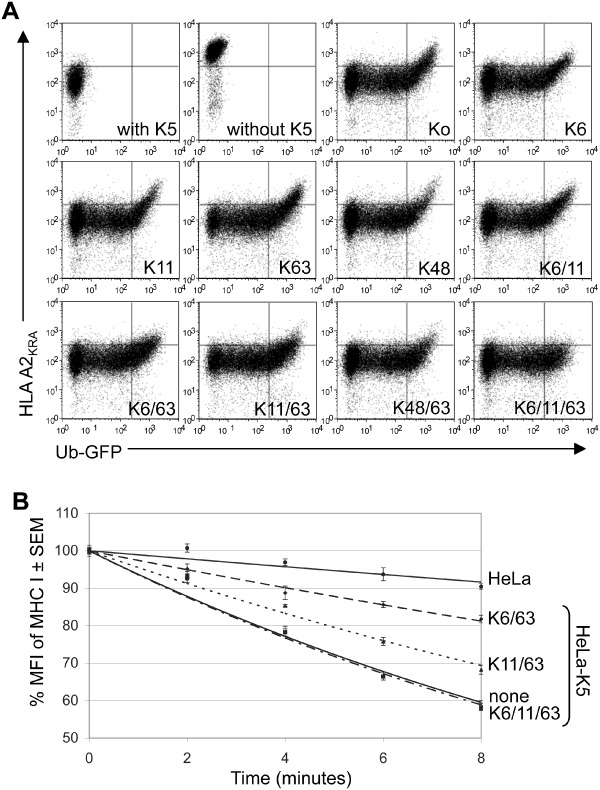
**Lys6, Lys11 and Lys63 of ubiquitin are necessary and sufficient for K5-mediated MHC I endocytosis.** A) K5 downregulation of MHC I with a single cytosolic lysine (K_335_) is restored by overexpression of a ubiquitin mutant expressing only three lysine residues (K_6/11/63_). Cytofluorometric analysis of HLA-A2_KRA_ expression in K 5+ cells 3 days post-transduction with ubiquitin mutants as noted. Cells were stained with BB7.2 + Cy5. B) Internalization assays (as in Figure 4B) of HeLa-K5 cells transduced with the KoUb-GFP-based mutants as noted. Mean of four replicates ± SEM.

Having established that Lys11 and Lys63 were necessary for K5-mediated downregulation of MHC I, we asked whether they were also sufficient. On the backbone of a lysineless ubiquitin mutant (KoUb-GFP), we replaced the indicated arginine residues back to lysines (K63) to determine which residues restored K5 function and prevented cell surface HLA-A2_KRA_ rescue (Figure 5A). As predicted, A2_KRA_ surface expression was rescued following Ko ubiquitin expression because polyubiquitin chains cannot be generated. Neither the single (Ko, K6, K11, K63, K48) mutants nor the double mutants (K11/63, K6/11, K6/63, K48/63) were sufficient to prevent a cell surface rescue of A2_KRA_ expression (Figure 5A). Only the triple mutant (K6/11/63) restored K5 function for both A2_KRA_(Figure 5A) and total MHC I (Figure S3), an effect confirmed with internalization assays (Figure 5B). Thus, Lys6, Lys11 and Lys63 of ubiquitin are necessary and sufficient for K5-mediated endocytosis of MHC I.

## Discussion

The role of ubiquitin in receptor-mediated endocytosis is well recognized, but the details remain unclear. We have used the ubiquitin E3 ligase K5 to dissect the polyubiquitin chain requirements for receptor internalization. Quantitative MS on MHC I immunoprecipitates identified an enrichment of MHC I-derived Lys11- and Lys63-linked polyubiquitin chains. It was not technically possible to perform MS analysis on single lysine expressing MHC I molecules, and MS analysis is unable to identify more than simple mixed ubiquitin chains. We therefore performed subsequent functional analyses using mutant ubiquitin constructs. Analysis of an MHC I (HLA-A2_KRA_) containing only a single lysine residue confirmed the requirement for both Lys11 and Lys63 linkages in receptor internalization. Therefore, our combined data from MS and mutant ubiquitin analysis demonstrate a requirement for Lys11 and Lys63 mixed linkage polyubiquitin chains in the K5-mediated downregulation of MHC I, and suggest a role for mixed linkage polyubiquitin chains *in vivo*.

Despite a recent quantitative proteomic analysis from yeast which reported Lys11-linked chains to be as abundant as Lys48 chains (5), the cellular role of Lys11-linked chains is not well understood. A role for Lys11 in receptor downregulation is not previously reported, although Lys11-linked ubiquitin chains are implicated in the regulation of cell cycle progression by the anaphase-promoting complex (APC/C)(27) and Lys11-linked chains selectively accumulate during ER stress (5).

**Figure 6:**
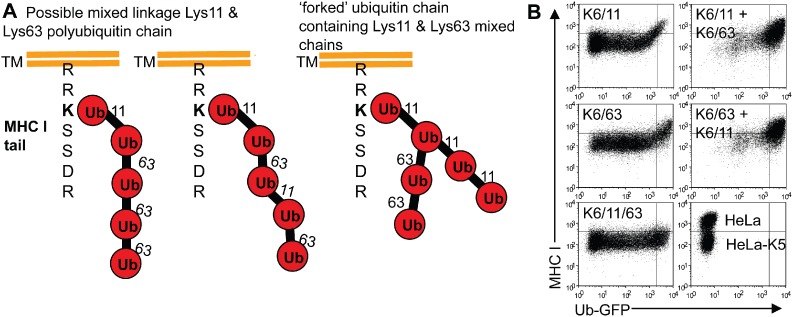
**Potential configuration of mixed linkage Lys11- and Lys63-linked polyubiquitin chains.** A) Mixed linkage chains can consist of non-homogeneous chains or forked chains as depicted. B) HeLa-K5 cells were transduced with a mixture of the K6/11 ubiquitin mutant to a GFP level sufficient to see a rescue of cell surface MHC I, and increasing concentrations of the K_6/63_ ubiquitin mutant. Conversely, starting with the K6/63 mutant and titrating in the K6/11 mutant also failed to restore K5 function, while the control K6/11/63 ubiquitin mutant fully supported K5-mediated downregulation of MHC I.

The requirement for both Lys11- and Lys63-linked chains in the K5-mediated downregulation of MHC I is difficult to understand. It is generally assumed that a polyubiquitin chain contains a single homogeneous chain linkage that determines the fate of the ubiquitinated substrate, but this assumption is difficult to confirm *in vivo*. As we identified lys-11 and lys-63 ubiquitin chains on endogenous MHC I by MS analysis, and confirmed their functional requirement by flow cytometry on a single MHC I lysine (K_335_) residue using HLA-A2 and ubiquitin mutants, our results imply a complex *in vivo* ubiquitin chain topology. A number of potential chain formations can explain these findings. The simplest model postulates the formation of a Lys11-linked chain between the first and second ubiquitin, with subsequent ubiquitin chain linkages involving Lys63 (Figure 6A). This might occur if polyubiquitin conjugation through Lys63 was conformationally unfavourable when the lysine acceptor on the substrate molecule is located close to the membrane, as is the case with K5-mediated ubiquitination of MHC I. Alternatively, the Lys63-linked polyubiquitin chain may be preassembled on the catalytic cysteine of the E2 enzyme before being transferred, *en bloc*, to the first ubiquitin acceptor on the substrate (28), and this linkage would require Lys11.

Other possible chain formations include a single ubiquitin chain with mixed Lys11 and Lys63 linkages, or a more complex ‘forked’ ubiquitin chain that contains two ubiquitins linked to a preceding ubiquitin (11,25) (Figure 6A). These forked chains can only be identified by MS when the ubiquitin moieties are linked to adjacent lysine residues on the ubiquitin, e.g. Lys6 + Lys11, or Lys29 + Lys33, but MS does not pick up forks from non-adjacent lysine residues as would occur with a Lys11 + Lys63 forked chain. While forked chains would have a requirement for Lys11 and Lys63 *in cis*, simple mixed chains would allow Lys11 and Lys63 chains to act *in trans* as well as *in cis*. Combined expression of the K6/63 and K6/11 ubiquitin mutants was unable to rescue the K5 phenotype (Figure 6B). This is consistent with a single lysine acceptor in the MHC I cytosolic tail, and suggests forked ubiquitin chains are more likely than simple mixed Lys11 and Lys63 ubiquitin chains.

Multiple ubiquitin chain linkages have been identified on polyubiquitin chains *in vitro.* For example, the complex polyubiquitination of cyclin B1 (25) did not affect ubiquitin receptor binding or degradation by the proteasome, whereas forked chain conjugations were resistant to isopeptidases and degraded poorly by the proteasome (11).

Little information is available on the use of mixed ubiquitin chain linkages *in vivo*. While endocytosis of the interferon-α receptor requires both Lys48- and Lys63-linked polyubiquitin chains for efficient internalization (29), endocytosis of the prolactin receptor requires Lys63-linked chains whereas the Lys48-linked chains identified in MS analysis were dispensable for internalization (9). With the interferon-α receptor, homotypic Lys48 and Lys63 chains on different lysine acceptor residues were seen, but could not be distinguished from mixed Lys48/Lys63 chains.

A requirement for polyubiquitin chains in the endocytosis of cell surface receptors is increasingly recognized, as also reported for the internalization of TrkA (7), aquaporin-2 (8) and MHC I (6). Our results confirm the absolute requirement for Lys63-linked polyubiquitin chains for MHC I endocytosis. MS analysis of ubiquitin chain linkages on denatured MHC I resulted in an enrichment for both Lys63 and Lys11. Despite an apparent functional requirement for Lys6 together with Lys11 and Lys63 for K5 function, no Lys6 ubiquitin chain linkages were detected in the MS analysis nor did the R6 mutant affect MHC I internalization. The proximity of Lys6 to the His68/Leu8 containing hydrophobic patch of ubiquitin suggests that mutation of this residue could contribute to MHC I rescue by perturbing binding by ubiquitin-binding proteins, including endocytic adaptors or other components of the ubiquitin proteasome system. Therefore, Lys6-linked ubiquitin chains are not essential for MHC I internalization, but Lys6 may either be important for recognition by adaptor molecules following the internalization of MHC I molecules (30) or for the structural integrity of ubiquitin.

For other receptors, Lys63-linked polyubiquitin chains appear to be required for sorting to the endosomal pathway rather than internalization. For example, Lys63-linked polyubiquitination of the yeast Gap1 permease is essential for entry into the multivesicular body pathway (31). In the case of the epidermal growth factor (EGF) receptor, monoubiquitylation may provide a sufficient signal for internalization, while Lys63-linked polyubiquitin chains are implicated in its targeting to lysosomes, rather than the initial internalization step (32). Finally, the lysosomal degradation of Deltex by Itch in the Notch signalling pathway is thought to be mediated by Lys29-linked polyubiquitin chains (33).

The structures adopted by different polyubiquitin chain linkages clearly affect function. Lys63-linked chains adopt a linear configuration, while a more compact structure is seen with Lys48-linked chains (34). For endocytosis, the open configuration of a Lys63-linked polyubiquitin chain increases avidity, which is thought to be necessary for binding the low-affinity ubiquitin-interacting motifs on the clathrin coat-associated sorting proteins (CLASPs) that link ubiquitinated cargo to clathrin (35). A mixed Lys11/Lys63 polyubiquitin chain may affect the avidity of the chain for CLASPS and explain the altered rate of endocytosis of MHC I in a HeLa-K5 versus a HeLa-K3 cell. Alternatively, as ubiquitination provides the signal for recognition and internalization of the ubiquitinated receptor, the identification of Lys11-linked ubiquitin chains predicts adaptors with specificity for these linkages, although these have not yet been identified.

We believe that the slower endocytosis of MHC I in the presence of K5 reflects two observations. First, the monoubiquitinated form of MHC I predominates in K5-positive cells and is a poor signal for internalization. We have calculated the percentage of mono- versus polyubiquitin chains from our radiolabelling experiments (Figure 1B). We find there is a threefold increase in monoubiquitinated MHC I as compared with the di-, tri- or tetraubiquitinated MHC I species, suggesting that polyubiquitination is indeed the rate-limiting step in K5-expressing cells. This differs from K3-expressing cells in which equivalent amounts of ubiquitinated species are seen on MHC I and very rapidly removed from the cell surface. Why is polyubiquitin chain extension rate limiting in a K5-expressing cell? K5 targets the membrane proximal lysine rather than the more distal lysine targeted by the K3 ligase. Perhaps due to the steric hindrance, extension of polyubiquitin chains on the ubiquitin attached to this membrane proximal lysine are rate limiting, and result in the extension of mixed Lys11/Lys63-linked polyubiquitin chains. We do not know the nature of the initial linkage, but suggest it may be Lys11. Therefore, the problems for K5 to extend polyubiquitin chains account for one of the reasons why MHC I is slowly internalized.

The second possible reason why MHC I is more slowly endocytosed in HeLa-K5 as opposed to HeLa-K3 cells is that the mixed Lys11/Lys63-linked chains catalysed by K5 are less well recognized by the endocytic adaptors than the pure Lys63-linked chains. Therefore, endoctosis is less efficient in a HeLa-K5 cell as compared with a HeLa-K3 cell. Our preliminary experiments with the ubiquitin mutants and their effects on K3-mediated downregulation of MHC I suggest no role for Lys11-linked chains.

Why would K5 have adopted this behaviour relative to K3? K5 appears to be more promiscuous than K3, downregulating a range of immunoreceptors. How it achieves this is unclear, but is likely because of its preference for the membrane proximal lysine –a common feature in the stop-transfer region of many membrane proteins. Furthermore, K3 is not expressed in the absence of K5. K5 is an immediate-early gene expressed throughout infection, while K3 is an early gene.

Despite a 49% peptide coverage, our MS analysis did not identify peptides from the MHC I cytoplasmic tail containing the lysine residue (K_335_) that serves as the ubiquitin acceptor site. The RKSSDR peptide may be too hydrophilic to bind to the C18 reverse phase column used, or too short to be detected by standard MS protocols. Peptide coverage did include the more distal lysine residue (K_340_) and as we had predicted, it was not ubiquitinated.

Our combined results suggest that a mixed linkage Lys11/Lys63 polyubiquitin chain is conjugated to the single lysine at position 335 on the cytosolic tail of HLA-A2 and that a single ubiquitin chain with complex topology is necessary for K5-mediated MHC I internalization. As further details on ubiquitin-dependent receptor internalization are uncovered, the increasing level of complexity matches the wide range of possible outcomes for target molecules. Some receptors need to be degraded while others unload ligand and are recycled. Providing subtly different signals, facilitated by different configurations of polyubiquitin chains, may allow fine tuning of the ubiquitin system and increased specificity. Further complexities in these signals will, no doubt, continue to emerge.

## Materials and Methods

### Cell lines

All cell lines were routinely grown in RPMI-1640 (PAA), 10% foetal bovine serum (PAA) and penicillin/streptomycin (SIGMA).

### Antibodies

The following antibodies were used: mAb w6/32, mAb HC10, mAb P4D1 anti-ubiquitin (Santa Cruz), mAb BB7.2 recognizes conformational HLA-A2 , MR24 (a kind gift of E. Wiertz) recognizes the ER luminal epitope of HLA, anti-ubc13 (Zymed), anti-ubcH5 (Boston Biochem), anti-calnexin (AF8 mAb) (a kind gift of M. Brenner), Cy5 and horseradish peroxidase (HRP)-conjugated secondary antibodies (Jackson).

### Lentivirus propagation, transduction and mutagenesis

The lentivirus expression system used was as described (22). The lentivirus expression plasmid vectors used were kind gifts of Y. Ikeda. Virus preparation and cell transduction have been described elsewhere (22). Site-directed mutagenesis (QuickChange®, Stratagene) was sequence verified.

### Flow cytometry

Samples were processed on a FACSCalibur as described (18) and analysed on flowjo software. The FL2 channel was used to analyse GFP expression in cells transduced with ubiquitin mutants.

### Internalization assays

Sixty to 72 h post-transduction with lentiviruses, cells were harvested by trypsinization and controls were set aside for Cy5 staining. The remaining cells were stained with w6/32 at 4°C and washed extensively with cold PBS. Samples were warmed to 37°C and at each time-point aliquots were harvested into cold PBS to stop endocytosis. Following the last time-point, all samples were stained with secondary antibody, and processed for flow cytometry. The geomean of w6/32 was determined and the data were analysed in Microsoft Excel. Starting levels of MHC I expression were normalized to 100% and the percent change in geomean (±SEM) expressed as a function of time.

### Radiolabelling, immunoprecipitation and polyacrylamide gel electrophoresis

Cells were pulse labelled for 10 min and chased with non-radioactive complete medium for 45 min. Cells were lysed and precleared of non-specific reactivity on protein A-agarose (Sigma) and/or immunoglobulin G (IgG) Sepharose (GE Healthcare) and then w6/32 or BB7.2 plus protein A-agarose was used for a primary IP of conformational MHC I. Following extensive washing, proteins were eluted from the protein A-agarose with 1% SDS in tris-buffered saline (TBS) pH 7.4 at 70°C for 10 min. The SDS was then sequestered with 10 v of 1% non-ionic detergent, the beads were spun out, and the supernatant was transferred to fresh tubes for the reIP with either HC10 (for the w6/32 primary IP) or MR24 (for the BB7.2 primary IP). To detect cell surface MHC I (Figure 1B), radiolabelled cells were washed and stained with w6/32 prior to detergent lysis. Postlysis, samples were split and either precipitated directly with protein A followed by HC10 reIP (S for surface MHC I), or additional w6/32 was added to precipitate the total w6/32 reactive MHC I pool (T for total MHC I). Samples were then subjected to SDS–PAGE and autoradiography. Cells used for MS analysis were harvested by scraping and lysed in a hypotonic buffer/freeze thaw procedure (36) in the presence of protease inhibitors. The membrane fraction was pelleted at 100 000 ×***g*** for 1 h and washed twice with 1 m sodium carbonate prior to solubilization with 1% SDS in TBS at 70°C for 15 min. The SDS was then sequestered with 10 v of 2% C12E9 (Calbiochem) in TBS pH8 and the solubilized membrane proteins were precleared of non-specific binding proteins with IgG Sepharose. MHC I was immunoprecipitated with HC10 (purified in house) covalently conjugated to protein A beads (Pierce Protein A IgG Plus Orientation Kit, Thermo Scientific), extensively washed in 0.1% C12E9 in TBS and eluted at 25°C for 5 min in the elution buffer supplied with the Pierce kit. Beads were excluded by centrifugation through Mobicol spin columns (MoBiTec) and the eluate was neutralized before loading buffer with DTT was added, samples were heated to 70°C and the products were separated on 9% polyacrylamide gels. The gels were fixed then stained with GelCode Blue Coomasie blue stain as per the manufacturer's instructions (Thermo Scientific). Gels were scanned and either dried (radioactive portion) and subjected to phosphorimager analysis, or bands were excised and stored at −80°C prior to MS.

### siRNA depletion

Ubc13 (D-003920-01, Dharmacon) and ubcH5c (6) siRNAs were reverse transfected into HeLa and HeLa-K5 using Dharmafect 1 (Dharmacon). Cells were incubated at 37°C/5% CO_2_ for 60 h prior to assay.

### Western blot analysis

Cells were lysed in 1% TX-100 in TBS plus protease inhibitors (Roche), and diluted into 1 × Laemli buffer prior to SDS–PAGE. Samples were transferred to polyvinylidene difluoride membranes (PVDF, Millipore), the membranes were blocked overnight at 4°C, then incubated with primary and secondary antibodies as noted. Membranes were developed in West Dura Extended Chemiluminescent substrate.

### Mass spectrometry

The proteins in the gel slices were digested by trypsin with the addition of eight stable isotope-labelled peptides as internal standards, including all seven −GG peptides and one ubiquitin peptide (Thr55-Lys63). The resulting peptide mixtures were analysed by reverse-phase LC-MS/MS on an LTQ-Orbitrap hybrid mass spectrometer. The instrument was operated to monitor the eight peptides and their related native counterparts by selective reaction monitoring (SRM). The quantification analysis was carried out using xcalibur software

We also applied protein identification with the same samples on the same LC-MS/MS platform. Peptide samples were eluted in a 60-min gradient with buffer B from 4 to 30% (buffer A, 0.4% acetic acid, 0.005% heptafluorobutyric acid and 5% acetonitrile; buffer B, 0.4% acetic acid, 0.005% heptafluorobutyric acid and 95% acetonitrile). The eluted peptides were detected in a precursor ion MS scan by Orbitrap after accumulation to a target value of 1 × 10^6^ in the linear ion trap (400–1600 *m*/*z*, 60 000 resolution at *m*/*z* 400, 1 µscan), followed by data-dependent MS/MS scans for the eight most abundant ions from each survey scan with an ion intensity above 500 counts and all decharged ions (isolation width of 2 *m*/*z*, 35% normalized collision energy, 1 µscan, target value of 5000, 60 seconds dynamic exclusion and preview mode applied). The acquired MS/MS spectra were searched with the Sequest-Sorcerer algorithm on a Sorcerer 2 IDA (Sage-N-Research) (37) against a composite target/decoy database to estimate the false discovery rate (38,39). The target proteins included human proteins supplemented with human ubiquitin sequence without methionine and common contaminant proteins, such as porcine trypsin and human keratins. The decoy proteins were generated from pseudo-reversed sequences of all target proteins. Searching parameters consisted of semitryptic restriction, and dynamic modification of oxidized or acetylated Met (+15.9949 and 42.011 Da, respectively). The initial mass search window was set to 20 ppm After that, the peptide matches were further filtered by dynamically increasing XCorr and ΔCn cutoffs until the global protein false discovery rate of 1% was reached. If the identified proteins only contained a shared peptide, they were merged into the same protein group.

## Supporting Information

Figure S1 K5 causes predominantly monoubiquitinated MHC Ispecies in a post-ER compartment. A) To test whether or not the high molecular weight class Ispecies detected, following MHC I IP were ubiquitinated heavychain, HeLa, HeLa-K3 or HeLa-K5 were subjected to radioimmuneprecipitation analysis. Following a 10-min pulse label and a 45-minchase, cells were lysed with TX-100 detergent and subjected toprimary IP with w6/32 followed by reIP with either HC10 (fordenatured class I heavy chain) or P4D1 (for ubiquitinated proteins)as indicated. Samples were then subjected to SDS–PAGE andautoradiography. B) To test if polyubiquitination occurs in the ER(EndoH sensitive) or a post-ER compartment (EndoH resistant),HeLa-K5 cells were subjected to radioimmune precipitation analysisas above except that following a 10-min pulse label, samples weretaken at the chase times indicated and each sample was subjected toEndoH digestion prior to gel electrophoresis.

Figure S2 Lys6, Lys11 and Lys63 of ubiquitin are necessaryfor K5-mediated downregulation of endogenous MHC I. K5 downregulation of endogenous MHC I is rescued by overexpression of the ubiquitin mutants R6, R11 or R63. Cytofluorometric analysis of MHC I expression in K 5+ cells 3 days post-transduction with ubiquitin mutants as noted. Cells were stained with W6/32 + Cy5 or Cy5 alone as a control.

Figure S3 Lys6, Lys11 and Lys63 of ubiquitin are sufficientfor K5-mediated downregulation of endogenous MHC I. K5 downregulation of endogenous MHC I is restored with a ubiquitin mutant containing Lys6, Lys11 and Lys63 and Arg at the other four positions. Cytofluorometric analysis of MHC I expression in K 5+ cells 3 days post-transduction with ubiquitin mutants based on a lysineless ubiquitin (Ko). Restoration of lysines is noted such that K6 has only a single lysine at position 6, while K48/63 has a lysine at positions 48 and 63. Cells were stained with W6/32 + Cy5 or Cy5 alone as a control.
