# Supplementary figures and images for: Efficient Internalization of MHC I Requires Lysine-11 and Lysine-63 Mixed Linkage Polyubiquitin Chains
Source: Traffic. 2009 Nov 17;11(2):210–20. doi: 10.1111/j.1600-0854.2009.01011.x (PMC3551259; doi:10.1111/j.1600-0854.2009.01011.x)

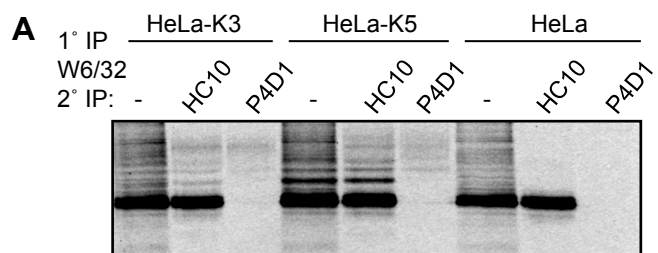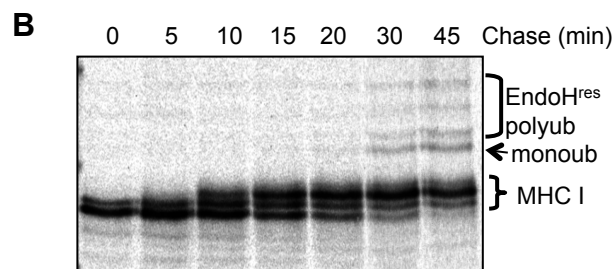

Supplement: Figure S1 — K5 causes predominantly monoubiquitinated MHC Ispecies in a post-ER compartment. A) To test whether or not the high molecular weight class Ispecies detected, following MHC I IP were ubiquitinated heavychain, HeLa, HeLa-K3 or HeLa-K5 were subjected to radioimmuneprecipitation analysis. Following a 10-min pulse label and a 45-minchase, cells were lysed with TX-100 detergent and subjected toprimary IP with w6/32 followed by reIP with either HC10 (fordenatured class I heavy chain) or P4D1 (for ubiquitinated proteins)as indicated. Samples were then subjected to SDS–PAGE andautoradiography. B) To test if polyubiquitination occurs in the ER(EndoH sensitive) or a post-ER compartment (EndoH resistant),HeLa-K5 cells were subjected to radioimmune precipitation analysisas above except that following a 10-min pulse label, samples weretaken at the chase times indicated and each sample was subjected toEndoH digestion prior to gel electrophoresis. [file tra0011-0210-sd1.pdf]

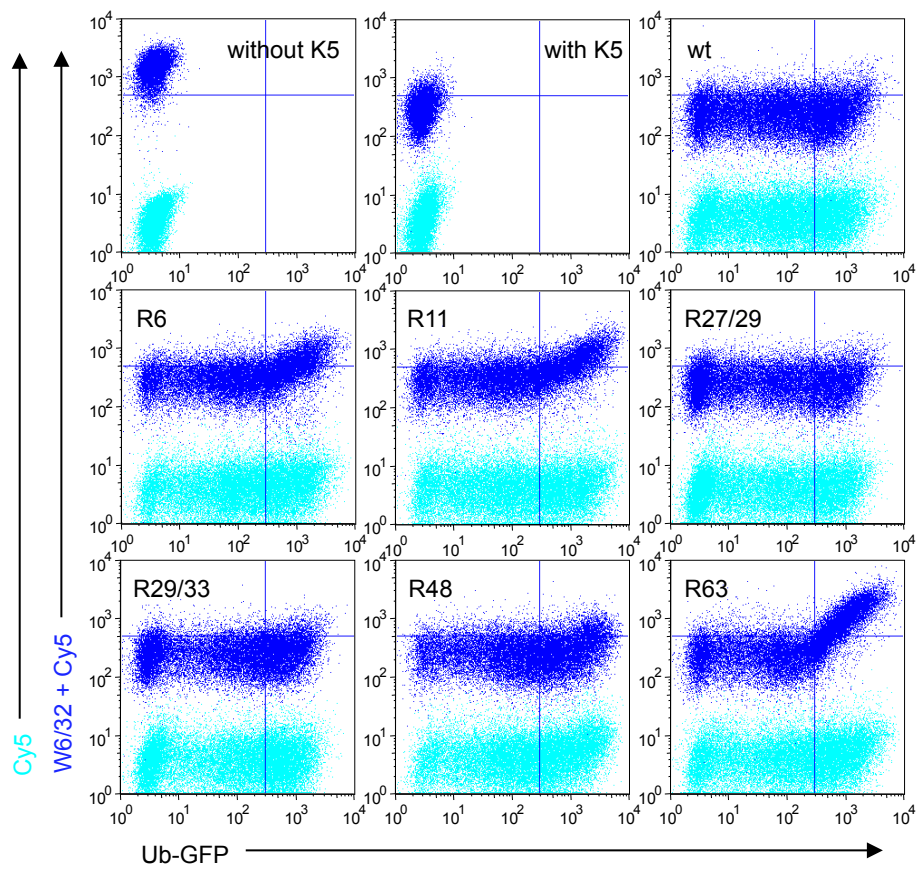

Supplement: Figure S2 — Lys6, Lys11 and Lys63 of ubiquitin are necessaryfor K5-mediated downregulation of endogenous MHC I. K5 downregulation of endogenous MHC I is rescued by overexpression of the ubiquitin mutants R6, R11 or R63. Cytofluorometric analysis of MHC I expression in K 5+ cells 3 days post-transduction with ubiquitin mutants as noted. Cells were stained with W6/32 + Cy5 or Cy5 alone as a control. [file tra0011-0210-sd2.pdf]

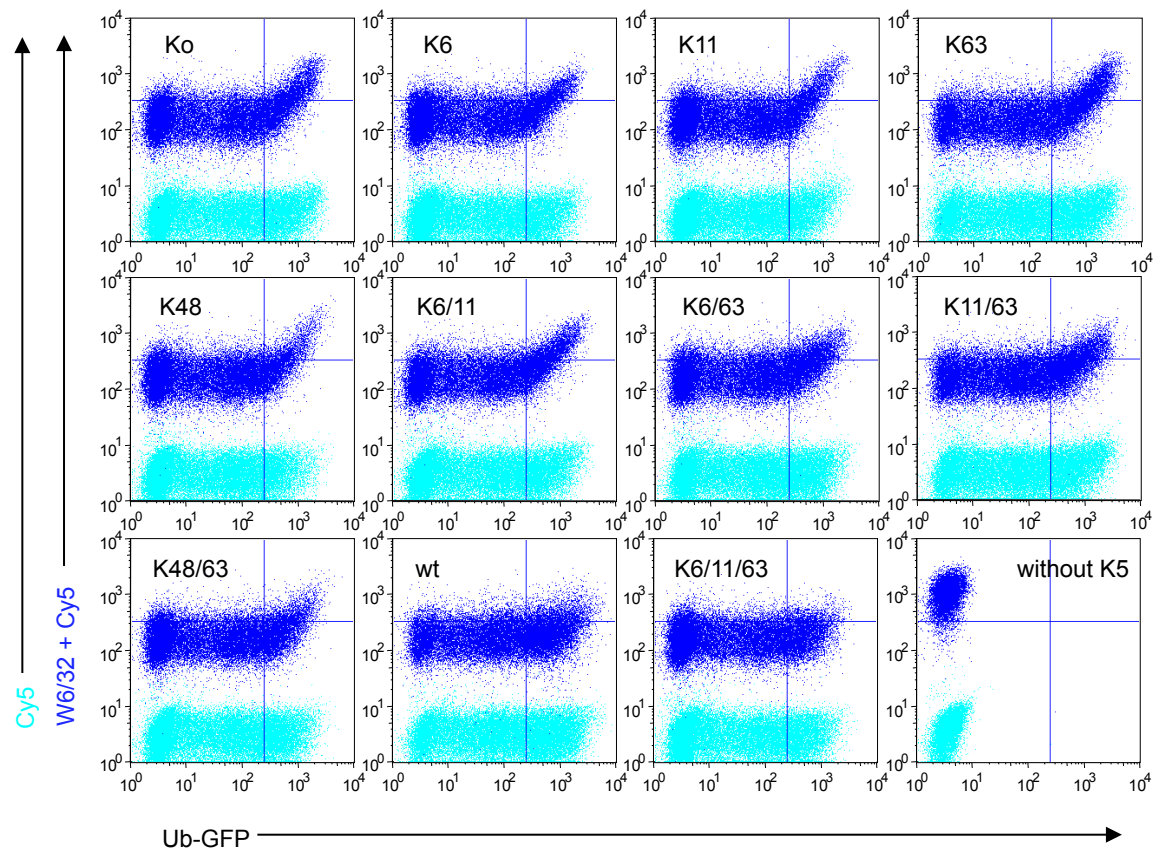

Supplement: Figure S3 — Lys6, Lys11 and Lys63 of ubiquitin are sufficientfor K5-mediated downregulation of endogenous MHC I. K5 downregulation of endogenous MHC I is restored with a ubiquitin mutant containing Lys6, Lys11 and Lys63 and Arg at the other four positions. Cytofluorometric analysis of MHC I expression in K 5+ cells 3 days post-transduction with ubiquitin mutants based on a lysineless ubiquitin (Ko). Restoration of lysines is noted such that K6 has only a single lysine at position 6, while K48/63 has a lysine at positions 48 and 63. Cells were stained with W6/32 + Cy5 or Cy5 alone as a control. [file tra0011-0210-sd3.pdf]
